# Evolution is coupled with branching across many granularities of life

**DOI:** 10.1098/rspb.2025.0182

**Published:** 2025-05-28

**Authors:** Jordan Douglas, Remco Bouckaert, Simon C. Harris, Charles W. Carter Jr., Peter R. Wills

**Affiliations:** ^1^ Physics, University of Auckland, Auckland, New Zealand; ^2^ Computer Science, University of Auckland, Auckland, New Zealand; ^3^ Max Planck Institute for the Science of Human History, Jena, Germany; ^4^ Statistics, University of Auckland, Auckland, New Zealand; ^5^ Biochemistry and Biophysics, University of North Carolina at Chapel Hill, North Carolina, USA; ^6^ Integrative Transcriptomics, University of Tübingen, Tübingen, Germany

**Keywords:** phylogenetics, Bayesian phylogenetics, molecular evolution, punctuated equilibrium, aminoacyl-tRNA synthetases, cephalopod, Indo-European, saltative branching, punctuated evolution, Beast 2

## Abstract

Across many scales of life, the rate of evolutionary change is often accelerated at the time when one lineage splits into two. The emergence of novel protein function can be facilitated by gene duplication (neofunctionalization); rapid morphological change is often accompanied by speciation (punctuated equilibrium); and the establishment of cultural identity is frequently driven by sociopolitical division (schismogenesis). In each case, the changes resist re-homogenization; promoting assortment into distinct lineages that are susceptible to different selective pressures, leading to rapid divergence. The traditional gradualistic view of evolution struggles to detect this phenomenon. We propose a probabilistic framework that constructs phylogenies, tests for saltative branching and improves divergence time estimation by estimating the independent contributions of gradual and abrupt change on each lineage. We provide evidence of saltative branching for proteins (aminoacyl transfer RNA (tRNA) synthetases), animal morphologies (cephalopods) and human languages (Indo-European). These three cases provide unique insights: for aminoacyl-tRNA synthetases, the trees are substantially different from those obtained under gradualist models; we estimate that 99% of cephalopod morphological changes coincided with speciation events; and Indo-European dispersal is estimated to have started around 6000 BCE, corroborating the recently proposed hybrid explanation. Our open-source code is available under a General Public License.

## 1. Introduction

*There are decades where nothing happens; and there are weeks where decades happen*.

Understanding the evolutionary relationships within groups of genes, species or cultures has traditionally hinged upon a fundamental assumption: that evolutionary change occurs independently of branching. Under the molecular clock hypothesis, evolution occurs in a clock-like manner, ticking through time at a more-or-less constant rate [1,2]. This view of evolution is embedded in phylogenetic clock models, including strict, relaxed and local [3–6]—each stemming from an assumption of extended, temporally regular, clock-like evolution or gradualism. This view cannot detect evidence for the scenario where evolutionary change is abruptly accelerated when one lineage splits into two or when the change is what causes the split. Rather, a phylogenetic tree is seen as a Jackson Pollock canvas that mutations are ‘thrown’ onto, in a manner that does not perturb the tree or alter the course of evolution. Application of an inappropriate phylogenetic model is often met with unwanted consequences; divergence time estimates can become biased, and unwarranted confidence can be placed in incorrect relationships [7,8].

**Figure 1.**
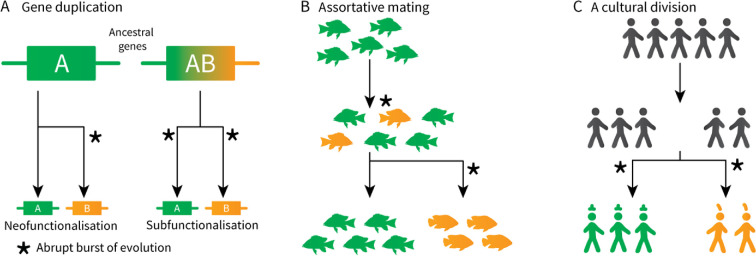
Evolutionary changes are often the driving force behind branching, or are accelerated by branching. The scenarios above depict examples of this process at molecular (A), morphological (B), and human cultural (C) scales. The coupling between evolutionary change and lineage splitting cannot be explained by clock-like evolution alone.

In reality, evolution is known often to occur in saltational leaps [9] that are frequently, but not necessarily, tied to branching (figure 1). In this scenario, the gradualistic view is dubious. We will refer to this apparent coupling between branching and abrupt change as *saltative branching*.

At the molecular level, gene duplication and lateral transfer can induce the *de novo* emergence of protein functions. In the 1930s, Haldane hypothesized that one copy of the gene would inevitably become silenced through random mutation [10]. This means that, conditional on both copies surviving, one paralogue (or both) is likely to have gained a novel selective advantage relative to the other. In some cases, one daughter copy evolves a novel function while the other retains its ancestral function (*neofunctionalization* [11]; figure 1A). In other cases, either daughter takes on a subset of the parental functions (*subfunctionalization* [12,13]). Such processes have given rise to a wide diversity of proteins, as exemplified by the spectrum of aminoacyl transfer RNA (tRNA) synthetases [14,15].

At the macroevolutionary level, Eldredge and Gould described a process they called *punctuated equilibrium* [16,17], whereby species undergo long periods of stasis characterized by gradual evolution, followed by short periods of abrupt evolution that are often linked to speciation. Although the theory of punctuated equilibrium was initially met with much skepticism and debate [18–20], it has since been quantitatively established in many systems: genetic [21–23], morphological [24,25] and even in cancer [26] and viruses [27]; while other times the hypothesis is rejected [28,29]. In some cases, branching accelerates the rate of species evolution, such as when a population migrates to a new habitat (adaptive radiation). In other cases, a sudden circumstantial change is what induces the branching, such as when individuals select mates with similar phenotypes (assortative mating [30]; figure 1B).

At the anthropological level, human cultures may change quite abruptly when new communities are founded. This creation of a social division has been termed *schismogenesis* by Bateson [31], and *esoterogeny* by Thurston [32]. When considering language evolution, the effect can manifest as rapid gains or losses of the *cognates* of a language (two words are considered cognate if they share common ancestry—for example ‘criatura’ in Spanish is cognate with ‘creature’ in English, but not ‘animal’). The notion of languages evolving like species—in punctuated bursts—has long been proposed as an important process in their development [32–35], and quantitatively demonstrated in several language families [36,37]. This might result from communities seeking to establish cultural identities (figure 1C), or as a founder effect—potentially in a new environment.

Saltative branching events share a structural similarity across these three cases. This similarity comes in three parts: a random heritable change, a foothold and a fixative event that drives population splitting. First, the change appears (e.g. mutation). Second, the environmental context, either internal or external to the system, provides a foothold allowing the change to produce a newly viable variant that persists through time and avoids re-homogenization. Third, this foothold permits a fixative process in which the change resists re-homogenization (and entrenches heterogeneity), and the assortment persists. The differential across the two lineages means they are susceptible to novel, or previously non-existent, unique selective pressures (e.g. new function or new environment), which will allow further random changes to accumulate and give the appearance of an accelerated clock rate; see *epistatic ratchet* [38]. The same pattern is seen in established models of the origin of life [39] and genetic coding [40]. Each is based on the splitting of initially homogeneous populations of either protocells or sets of proteins, respectively, initiated by the growth of a fluctuation (random change) away from a homogeneous ground state, through a dynamic instability where the bifurcation establishes a foothold, leading to its fixation in a new heterogeneous state.

**Figure 2.**
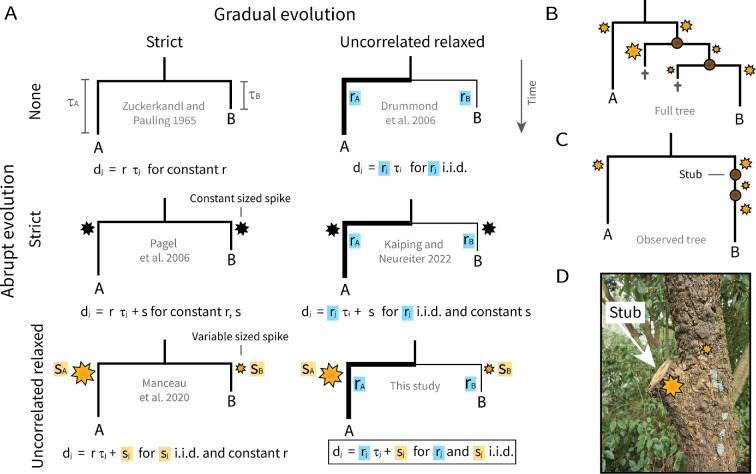
Evolutionary clock models. (A): the total evolutionary distance of a branch d is a function of time τ, clock rate r, and spike s. In the relaxed models of gradual evolution, branch rates (line widths) are assumed to be independent and identically distributed (i.i.d.); coloured blue. The relaxed models of abrupt evolution assume that each branch is characterized by an instantaneous spike of evolution (stars), and the sizes of those spikes are also, i.i.d.; orange. Note that Manceau *et al*. [23] assumed that branch spikes take a more restricted range of values, rather than the continuous magnitudes depicted here. Correlated or local clock models represent a midpoint between strict and uncorrelated. (B-C): if one assumes that each branch is associated with an evolutionary spike, then unobserved speciation events (i.e. stubs) must be accounted for. These unsampled taxa may leave behind a footprint on those that were sampled by inducing now-hidden evolutionary bursts. (D): a stub on a wooden tree at the University of Auckland.

These cases highlight the inability of gradualism to explain important evolutionary processes. Phylogenetic clock models—such as strict [1], relaxed [3,4,41] and local [4–6,42]—describe ways in which change occurs on average through time (figure 2). Strict clocks impose a fixed rate of change, while relaxed (uncorrelated) clock rates vary independently across lineages. ‘Relaxed clock’ is usually shorthand for ‘relaxed gradual clock’, but abrupt changes at branch points might also be strict (with each branch experiencing an instantaneous ‘spike’ of constant magnitude [21,37]) or relaxed (with each branch having a spike of independent magnitude [23]). The term ‘instantaneous’ is of course time-dependent—a day is just an instant on the scale of millennia, and in this context describes enough time for evolutionary changes to accrue. If the branches of the tree represented short time intervals (e.g. single generations), then a spike in evolutionary rate might in fact span several branches and not just one. Owing to the comparatively slow rate of evolution, the model we describe here, which assigns spikes to each branch independently, might no longer be appropriate in this scenario.

To investigate the link between branching and change, we provide a probabilistic method for building phylogenies and testing abrupt evolution from discrete character data, such as molecular sequences, morphological traits, or cognates. Expanding on existing methods [21,23,37,43], our model captures both abrupt and gradual evolution, with either process relaxed, meaning they can freely vary among branches. Unlike strictly gradual models, here, even the shortest of branches may still harbour significant levels of evolutionary change. If branching and evolution are indeed tightly coupled, then unobserved speciation events may have left behind a footprint on the lineages that have been observed [23,37,43]. We assume that each lineage experiences a rapid evolutionary spike, the size of which is informed by the number of unobserved bifurcations along that branch (i.e. the number of *stubs*; figure 2). These stubs result from lack of sampling, perhaps owing to extinction, cryptic species or other forms of missing data.

## 2. Methods

We will demonstrate what is gained by considering the effects of abrupt evolution in addition to gradual change. Gradualistic divergence can be thought of as independent diffusion, and abrupt change as a transient repulsion that drives the lineages apart even further. As shown in figure 2A (bottom right), each branch consists of a length (time), a rate (gradual evolution) and a spike (abrupt evolution). Using discrete character data, we will estimate these terms, as well as the tree topology and other parameters. These will be estimated using Bayesian phylogenetic inference, by sampling from the posterior probability distribution of trees and parameters. We will test the hypothesis of saltative branching by benchmarking the proposed ‘gradual+abrupt’ clock model (with both components relaxed) against a standard ‘gradual’ relaxed clock model [3,41]. The alternatives are compared using Bayesian model averaging [44], by estimating the clock model indicator as if it were a parameter; $I_{c}=0$ is the gradual clock, and $I_{c}=1$ is gradual+abrupt. Much like the Akaike and Bayesian information criteria, Bayesian model averaging penalises overparameterized models, and is therefore expected to disfavour our more elaborate spike model when it has little to offer.

We will first formalize our branching process in order to model the number of stubs along each branch. Then, we will show how these stubs are used to assign each branch an evolutionary distance.

### (a) Probability density function of a tree and its stubs

Let T be a binary-rooted time-tree. We will assume that branching in T follows a fossilized birth-death process, as described by Stadler [45]. In this model, a lineage will either branch into two (at rate λ) or become extinct (at rate μ). Ancestral lineages are independently sampled at rate ψ, while extant lineages are sampled with probability ρ. In contrast to previous approaches that are often employed in epidemiology [46], here we assume that sampling an ancestral lineage does not ‘remove’ the lineage from the tree. We will also assume sub or supercritical conditions, i.e. λ≠μ.

We will describe time in the reverse direction, i.e. age or height. Suppose that T contains n extant nodes (at age 0), m extinct nodes (at ages y>0) and k sampled ancestors. There are n+m−1 internal nodes (with ages x>0), including the root at age $x_{1}$. As described by eqn (9) of [45], the probability density of observing tree T (conditional on n) is:


(2.1) $$p(T|\lambda ,\mu ,\psi ,\rho )=\frac{4n\rho \lambda \psi^{k+m}}{c_{1}\left(c_{2}+1\right)\left(1-c_{2}+\left(1+c_{2}\right)e^{c_{1}x_{1}}\right)}\times \prod_{i=1}^{n+m-1}\lambda p_{1}\left(x_{i}\right)\prod_{i=1}^{m}\frac{p_{0}\left(y_{i}\right)}{p_{1}\left(y_{i}\right)},$$

where $p_{0}(t)$ and $p_{1}(t)$ are the probabilities of an individual at age t respectively having 0 and 1 sampled descendants:


(2.2) $$\begin{array}{ll} p_{0}(t)=\frac{\lambda +\mu +\psi +c_{1}\frac{\left(1-c_{2}\right)e^{-c_{1}t}-\left(1+c_{2}\right)}{\left(1-c_{2}\right)e^{-c_{1}t}+\left(1+c_{2}\right)}}{2\lambda } & c_{1}=\left|\sqrt{(\lambda -\mu -\psi {)}^{2}+4\lambda \psi }\right|\\ p_{1}(t)=\frac{4\rho }{2\left(1-c_{2}^{2}\right)+e^{-c_{1}t}{\left(1-c_{2}\right)}^{2}+e^{c_{1}t}{\left(1+c_{2}\right)}^{2}} & c_{2}=-\frac{\lambda -\mu -2\lambda \rho -\psi }{c_{1}} \end{array}.$$

Now, let F be the full phylogeny of our observed tree T, before sampling has occurred (figure 2B,C). That is, F contains all of the leaves in T, plus additional extant and ancestral lineages that were not sampled. Following the reduced tree decomposition (e.g. section 3.3 of Harris *et al.* [47]), let us define a *stub* as an internal node in F, conditional on one child lineage being sampled in T and the other being unsampled (figure 2). Note that the k sampled ancestors do not count as stubs, because they do not correspond to bifurcation events in F. Stubs appear at a time-dependent rate $2\lambda p_{0}(t)$ in T. Following eqn (1) of Stolz *et al.* [48], the expected number of stubs along an interval $\left[t_{o},t_{e}\right]$ is:


(2.3) $$E(B)=\int_{t_{e}}^{t_{o}}2\lambda p_{0}(t)dt=\left(t_{o}-t_{e}\right)\left(\lambda +\mu +\psi -c_{1}\right)+2\log\left(\frac{\left(c_{2}-1\right)e^{-c_{1}t_{e}}-\left(1+c_{2}\right)}{\left(c_{2}-1\right)e^{-c_{1}t_{o}}-\left(1+c_{2}\right)}\right).$$

**Figure 3.**
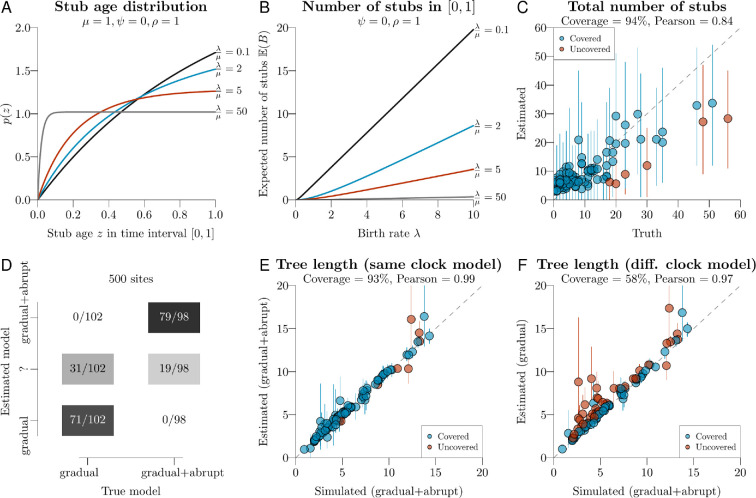
Characterizing the probabilistic properties of the model. (A): most stubs occur near the start of a lineage. Intuitively, the probability that an older lineage will go extinct before the present day (and creating a stub) increases with the age of that lineage. Thus, stubs are more likely to occur earlier in time. (B): the number of stubs along a lineage depends on branching parameters: birth rate λ, death rate μ, historical sampling rate ψ, and contemporaneous sampling probability ρ. (C): coverage simulation study for estimating the number of stubs. Each point is one Markov chain Monte Carlo (MCMC) chain and one simulated set of parameters; mean estimates are depicted by circles, and 95% ~credible intervals by vertical lines. (D): the true clock model can be recovered from simulated data; the middle row `?' denotes the ambiguous case when the inferred model has a Bayes factor of less than 10 (i.e. $0.09<p(I_{c}=1)<0.91$). E–F: each point is a dataset simulated under the gradual+abrupt clock model, with inference done under either gradual+abrupt (E) or gradual (F). Divergence times are being overestimated in the latter.

The number of stubs B follows a Poisson distribution with rate E(B). Given that stubs are conditional on one lineage being unsampled, they are more likely to occur further back in time (figure 3A). The probability density of observing B stubs at ages $Z=\left(z_{1},z_{2},\ldots \;,z_{B}\right)$ along interval $\left[t_{o},t_{e}\right]$ is then:


(2.4)p(Z,B|to,te,λ,μ,ψ,ρ)=e−E(B)B!∏j=1B2λp0(zj).

Lastly, let $B_{i}$ and $Z_{i}$ be the number and ages of stubs on branch i, and let $x_{e_{i}}$ and $x_{o_{i}}$ be the ages of branch i and its origin. Then, the overall probability density of observing a tree and its stubs is:


p(T,Z,B|λ,μ,ψ,ρ)=p(T|λ,μ,ψ,ρ)×p(Z,B|T,λ,μ,ψ,ρ)(2.5)=p(T|λ,μ,ψ,ρ)×∏i=22(m+n+k)−1p(Zi,Bi|xoi,xei,λ,μ,ψ,ρ).

The product in the above equation starts at i=2, since i=1 corresponds to the root, and here we assume for simplicity that there are no stubs on the root branch (the origin). The number of stubs, their ages, and their branch indices can either be either estimated directly or integrated out of the model (electronic supplementary material, S2).

### (b) Clock models

Each internal branch i in T is associated with a time duration $\tau_{i}$, a relative rate $r_{i}$ and a spike $s_{i}$ (figure 2). Spike sizes are independent of time units, for example a spike of size 0.1 means that around 10% of sites in the alignment are expected to change at the bifurcation, regardless of time units. The total evolutionary distance of a branch is then $\mu_{c}\times \tau_{i}\times r_{i}+s_{i}$, where $\mu_{c}$ is the clock rate. These distances are used to compute the tree likelihood using Felsenstein’s peeling algorithm [49].

Under the relaxed model of gradual evolution, each relative rate is independent and identically distributed with a mean of 1 so that it does not conflate with $\mu_{c}$ [3,41]:

.(2.6)ri∼LogNormal(μ=−σr22,σ=σr).

The standard deviation $\sigma_{r}$ should be reasonably small (e.g. <0.5) to ensure that these rates are constrained. This constraint is enforced by our prior distribution on $\sigma_{r}$. We use the real-space rate parameterization of this model (implemented in the BEAST 2 ORC package [41,50]), which is built on top of the classic discretized form by Drummond *et al*. [3].

Under the relaxed model of abrupt evolution, the total sum of spike sizes on a branch is dependent on the number of stubs along that branch. These stubs correspond to clades that were not sampled, owing to extinction or otherwise lack of observation, but their existence is assumed to have left behind a trace on the branch that did get sampled. Therefore, the total number of spikes on a branch is equal to the number of stubs along that branch, plus one (corresponding to the parent node). If there are no stubs in the tree (for example owing to a high sampling rate or a low extinction rate), then each branch has just one spike.

This model has two hyperparameters: the spike mean $S_{\mu }$ and the spike shape $S_{\alpha }$. Each spike is independent and identically distributed under a gamma distribution with shape v and scale w:


(2.7)si=IcSμsi′, where si′∼Γ(v=Sα(Bi+1),w=1Sα),

where $B_{i}$ is the number of stubs on branch i, and $I_{c}\in \left\{0,1\right\}$ is the clock model indicator, which determines whether we are using the gradual ($I_{c}=0$) or gradual+abrupt ($I_{c}=1$) model. This latter assumes that each bifurcation contributes an independent spike whose size is Gamma($S_{\alpha },1/S_{\alpha }$) distributed. The total branch spike sum $s_{i}$ is estimated rather than per-stub. Keeping the mean $S_{\mu }$ outside of the Gamma distribution (which has a mean of 1) helps to overcome numerical and mixing issues. Analogous to the standard deviation of the gradual relaxed clock, the shape $S_{\alpha }$ should be large (e.g. >0.8), and therefore the variance small, so that spike sizes are constrained. Based on our estimates of spike size here, as well as previous ones [21,23,37], we recommend using an $S_{\mu }$ prior with a mean of around 0.01 in the general case where no information is known, e.g. LogNormal(mean = 0.01, σ=1.2).

The total proportion of evolutionary change that can be explained by saltative branching is quantified by SB. Note that nodes with branch length zero are omitted from this calculation, i.e. sampled ancestors and the root:


(2.8)SB=∑i=2n+m−1(si)÷∑i=2n+m−1(riτiμc+si).

### (c) Bayesian phylogenetic inference

Combining the gadual and abrupt clock models with the fossilized birth-death tree prior plus stubs, the overall posterior density is:


p(T,Z,B,λ,μ,ψ,ρ,Sμ,Sα,μc,s,r,σr,Ic|D)⏞posterior∝p(D|T,θs,s,r,μc,Ic)⏞tree likelihood×p(r|σr)⏞branch rate prior×p(s|Sμ,Sα,Z,B)⏞spike prior×p(T,Z,B|λ,μ,ψ,ρ)⏞stumped tree prior(2.9)×p(Ic)p(θs)p(Sμ)p(Sα)p(σr)p(μc)p(λ)p(ψ)p(μ)p(ρ)⏞hyperpriors

where $\theta_{s}$ contains parameters related to the site model (such as transition-transversion ratio in the case of DNA data, or amino acid frequencies in the case of protein). In all cases here, the clock model indicator has a $I_{c}∼\text{uniform}(\{0,1\})$ prior. This posterior distribution is sampled using Markov chain Monte Carlo (MCMC). As outlined in the electronic supplementary material, we built upon an advanced pool of MCMC proposal kernels [41,46,50–53] to ensure an efficient traversal of the posterior distribution, including tree topology, branch lengths, sampled ancestors and stubs.

**Figure 4.**
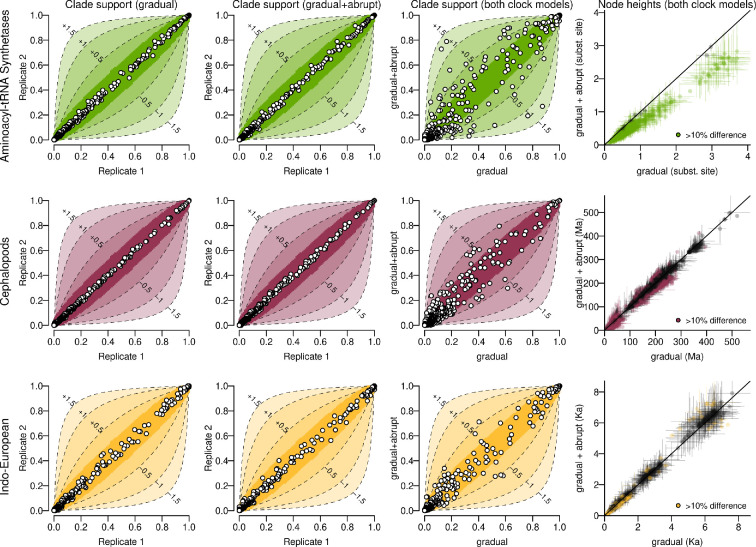
Each point is a clade found in the posterior distribution of one or both MCMC chains. Columns 1−3: clade supports are shown on *cocoa-fruit plots*. The first two columns depict two independent MCMC replicates, whose clade supports should lie close to the diagonal if the two chains have indeed converged to the same distribution, shown by the statistically insignificant zone (inner contour). The third column compares both clock models. Many clades are associated with substantial changes in log Bayes factor between the two models (outer contours). We refer the reader to the electronic supplementary material, S6 for further details on these contours. Column 4 compares node heights estimates across the two clock models; points are means and lines are 95%~credible intervals.

The support of a clade is estimated as the number of times that clade was sampled in the posterior distribution, divided by the total number of samples. These supports are displayed as cocoa-fruit plots (figure 4), where any clade that is observed in just one of the two MCMC chains is assigned a zero probability in the latter, noting that many of these clades are subsets of other displayed clades.

The inner (dark colour) and outer (light colour) contours on figure 4 have different meanings, as detailed in the electronic supplementary material, S6. In brief, the inner contours denote the statistically insignificant zones of clade support that comes from sampling error; while the outer contours describe difference in log Bayes factor [54] of the clade, with larger differences representing more extreme discrepancies between the two tree distributions.

## 3. Results

### (a) Simulation studies

The gamma spike clock model was implemented in a probabilistic setting. In this way, parameters, trees, and sequences can be directly simulated under a model the same or different from, that used during inference. We used this property to validate our new method through coverage simulation studies [55]. This was achieved by: (i) sampling parameters θ and a tree T from a probability distribution; (ii) simulating a multiple sequence alignment D under θ and T; (iii) performing MCMC on data D to obtain estimates $\hat{\theta }$ and $\hat{T}$; and lastly (iv) comparing θ with $\hat{\theta }$, and T with $\hat{T}$. These four steps were repeated multiple times. In this case, θ includes various parameters related to the site model, branching process, and clock model; including the clock model indicator $I_{c}$ (detailed in the electronic supplementary material, S4). When the model is correctly implemented, one would expect the true value of a parameter to reside in its 95% credible interval in approximately 95% of all replicates (in which case we say it has 95% coverage). These experiments confirmed the usefulness and correctness of the method, whose estimates were well-correlated with the true values and had around 95% coverage (electronic supplementary material and figure 3C).

Second, we evaluated the method’s ability to identify the true clock model using Bayesian model averaging, on a range of dataset sizes. To do this, we simulated more datasets under either model (gradual vs gradual+abrupt) and estimated the clock model indicator $I_{c}\in \left\{0,1\right\}$. A Bayes factor of 10 indicates ‘strong’ support in favour of one hypothesis over another [54], corresponding to $p(I_{c}=1)=0.91$ (or 0.09) when the two hypotheses are equal *a priori*. Using this threshold, and ‘true’ spikes averaging 0.01 substitutions per site per bifurcation, the method recovered the true clock model in 150 out of 200 simulated alignments (with 500 sites) and was uncertain in the remaining 50 out of 200 (figure 3D). On smaller datasets with just 20 and 100 sites, the clock model was correctly identified 13 out of 200 and 81 out of 200 times respectively, showing that accuracy improves with increasing data volume. The wrong model was never selected. We also assessed the effect of model misspecification—where the prior distributions used during inference differed vastly from those used during simulation. Even under these conditions, the clock model was still rarely wrong, and support in favour of the correct model grew with increasing alignment length (electronic supplementary material, figure S4). These results are reassuring; they confirm the method (i) is statistically consistent, (ii) is reliable at hypothesis testing even when there is no prior information, and (iii) does not have a proclivity to ‘overfit’ using the extra parameters of the larger model.

Third, we assessed the effect of model misspecification on divergence time estimation (figure 3E,F). To do this, we estimated divergence times for each clock model on datasets that were simulated under the gradual+abrupt model. The 100 trees used here were again serially sampled time-trees. The sequence alignments (with 100 sites) were simulated with an average spike size of 0.02 substitutions per site per bifurcation (abrupt evolution), and an average clock rate of one substitution per site per unit of time (gradual evolution). We then compared the known tree length (i.e. the sum of all branch lengths) with the tree length inferred under either clock model (gradual vs gradual+abrupt). Unsurprisingly, the gradual+abrupt model was able to infer the correct tree length (figure 3E), whereas branch lengths (time) were overestimated (by 16%) under the gradual model (figure 3F). By contrast, we did not observe any significant impacts on the topology of the summary tree in our simulation studies (electronic supplementary material, S4.4).

### (b) Empirical datasets

We assessed these two views of evolution on 14 empirical datasets. First, we screened the method on a widely used collection of 11 nucleotide datasets DS1−11 [56]. Using a Bayes factor threshold of 10, five of these datasets favoured the gradual+abrupt model, three rejected it, and the remainder were less certain. The datasets that supported the hypothesis of saltative branching yielded mean spike sizes $S_{\mu }$ ranging from 0.005 to 0.02 changes per site per bifurcation, which explained from 9 to 66% of all evolutionary changes i.e. SB (electronic supplementary material, table S1). The datasets that rejected the model yielded much smaller estimates for these two terms.

**Figure 5.**
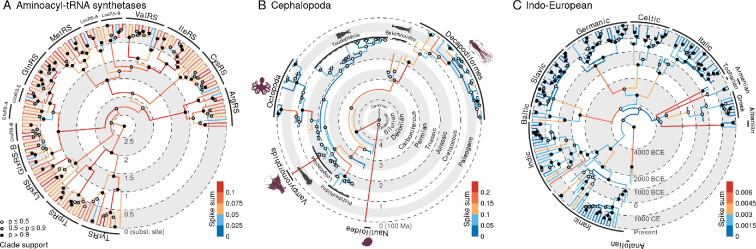
Phylogenies inferred under the gradual+abrupt clock model. The three-point estimates summarize their respective tree posterior distributions using the conditional clade distribution 0 method [57]. Median estimates of branch spike sums are shown.

Second, we took a deeper dive into three empirical case studies: aminoacyl-tRNA synthetases (protein sequences), cephalopods (animal morphologies) and Indo-European languages (cognates). The gradual+abrupt evolution model was overwhelmingly favoured, with estimated probability $p(I_{c}=1)=1$ in each case, suggesting that these systems underwent significant rapid changes at the time of branching. The trees in figure 5 summarize the gradual+abrupt tree posterior distributions shown in figure 4, which reveal that the competing models yielded divergent clade probabilities, to statistically significant and often substantial levels. This effect was strongest for the protein dataset. As shown in table 1, the average spike size varied considerably between the three datasets, from 0.001 substitutions per site per bifurcation for the language dataset, to 0.09 for the proteins. Although a spike size of 0.001 seems rather small, this term translated to SB = 0.24, meaning that 24% of all evolutionary change occurred at the branch points in the Indo-European dataset. This figure is 27% in the proteins, and a remarkable 99% for the cephalopods. This resulted in the estimated gradual clock rates being lower (with the exception of the protein dataset, whose clock rate was fixed owing to the absence of temporal calibration). The data provided no signal for estimating the shape of the spike Gamma distribution $S_{\alpha }$.

**Table 1.** Comparison of the clock models, gradual (G) and gradual+abrupt (G+A), on three empirical datasets. (Parameter mean estimates (and 95% credible intervals) are shown. Tree heights and lengths are in units of substitutons per site (aaRS), Ma (cephalopods) and Ka (Indo-European). Therefore, clock rates are, respectively, in units of amino acid substitutons per site, changes per trait per million years, and changes per cognate per thousand years. $\rho_{sr}$ is the average Pearson correlation between the estimated spike and rate of each branch, and SB is the total proportion of evolutionary change that is explained by saltative branching across the entire tree equation(2.8)).

dataset	clock model	tree height	tree length	clock rate $\mu_{c}$	spike mean $S_{\mu }$	spike shape $S_{\alpha }$	stubs	$\rho_{sr}$	SB
aaRS	G	3.69 (3.07, 4.41)	111 (102, 120)	1	—	—	14.9 [1,33]	—	0
	G+A	2.83 (2.14, 3.57)	79.3 (62.9, 94.9)	1	0.088 (0.046, 0.13)	1.7 (0.76, 3)	14.9 [2,33]	−0.12	0.27
cephalopods	G	494 (469, 553)	3790 (3210, 4370)	0.0044 (0.0022, 0.0071)	—	0	186 [85,296]	—	0
	G+A	500 (469, 567)	3680 (3070, 4310)	2.2e−05 (7.0e−09, 9.2e−05)	0.045 (0.025, 0.067)	2.3 (0.96, 4)	111 [60,163]	−0.00081	0.99
Indo- European	G	7.62 (5.99, 9.35)	186 (164, 207)	0.011 (0.0066, 0.017)	—	—	188 [138,241]	—	0
	G+A	7.79 (5.94, 9.75)	183 (159, 206)	0.0095 (0.0046, 0.018)	0.0012 (0.00047, 0.0023)	2 (0.85, 3.6)	190 [136,241]	−0.17	0.24

### (c) Protein study: aminoacyl-tRNA synthetases

The aminoacyl-tRNA synthetases (aaRS) are a large group of enzymes that implement the genetic code by attaching amino acids to their cognate tRNAs [58]. The complete set of aaRS families, which enable the coding of 20-plus canonical amino acids, emerged from a series of ancient structural and functional diversification events that can be traced back to the earliest stages of life on Earth before the last universal common ancestor [14,15]. Therefore, it is unsurprising to find that so much of their evolution has occurred in sudden bursts, previously described as quasi-species bifurcations [59].

We considered a previously published curated alignment of aaRS three-dimensional structural models [60], consisting of 494 amino acid sites from 142 Class I aaRS catalytic domains sourced from bacteria, archaea, eukaryotes, and viruses. The two clock models produced substantially different clade posterior supports (figure 4), which were not only statistically significant, but also induced large changes to clade Bayes factors. As shown in table 1, these proteins have undergone considerable mutational saturation over their presumed four billion years of evolution, with an estimated tree height of around 3.7 gradual substitutions per site. The gradual model overestimated the tree height by around 30%, relative to the gradual+abrupt model, reflecting the large mutational spikes, which saw one in every 11 amino acids change at each bifurcation. As shown in our phylogenetic tree (figure 5), most lineages, young and old, were associated with sizeable spikes, notably those that gave rise to aaRS families; including arginyl-tRNA synthetase (ArgRS), lysyl-tRNA synthetase (LysRS) and isoleucyl-tRNA synthetase (IleRS). The relationships between the families are similar to those of our previous tree, which additionally took structural insertion modules into account [15]. An even more reliable phylogeny may be obtained by accounting for both abrupt evolution and the gain of insertion modules. It is noted that this analysis still makes the critical assumption of the amino acid alphabet being fixed to 20 characters through time; an assumption violated by the translational reflexivity unique to aaRS synthesis [59].

### (d) Morphology study: cephalopods

The Cephalopoda are a class of molluscs characterized by their soft bodies and variable numbers of arms and tentacles [61]. The ingroup (Coleoidea) showcases advanced intelligence and mimicry skills [62,63] and includes the ten-armed Decapodiformes (squids and cuttlefish), and the eight-armed Octapoda (octopuses) and Vampyromorphida (vampire squid; *Vampyroteuthis infernalis*). The outgroup (Nautiloidea) consists of a primitive group of molluscs, often regarded as ‘living fossils’ [64], with a coiled shell and up to a few dozen appendages.

Whalen & Landman [65] compiled a dataset of cephalopod morphological traits, detailing both living organisms and fossils. These 153 discrete traits describe shell shapes, tentacle structures, the number of fins, and various other properties of 27 living cephalopods and 52 fossils. In contrast to the aaRS case, the data characterizing these taxa contains temporal information; the oldest taxon being a fossilized *Nautilus Pompilius* specimen dated at 469 million years ago. The authors used this dataset to build a phylogenetic tree under a fossilized birth-death tree prior and a strict (gradual) clock model.

Using the same dataset, we found that gradual evolutionary clock rate estimates varied by two orders of magnitude between the two models, with 99% of all evolutionary change occurring on the nodes (table 1). Gradual evolution played a trivial role in morphological evolution with just 1.7 of 153 traits estimated to change over 500 million years through gradualist diffusion, but an average of 6.9 of 153 changes per speciation event. Hence, the ‘gradual+abrupt’ model is effectively more of an ‘abrupt’ model in this case. As a result, the spikes and rates on each branch were uncorrelated $\left(\rho_{sr}\approx 0\right)$. The two clock models also yielded different clade probabilities, at a statistically significant level, but the differences are not as extreme as for the aaRS (figure 4). As shown in figure 5, there are two lineages associated with disproportionately large levels of abrupt change: the lineages that gave rise to (i) the coleoid ingroup during the late Cambrian period (average spike sum: 0.37 (0.08, 0.76)), and (ii) the vampire squid during the Jurassic period (0.31 (0.13, 0.57)). We note that the large spike sum on the nautoloid outgroup lineage is a false positive reflecting the large number of stubs required to account for the putative evolutionary relationship between the outgroup and the Coleoidea ingroup; in reality this living specimen has the same morphology as its fossilized ancestor near the root of the tree. This work further corroborates the phylogeny produced by Whalen & Landman [65], and is generally consistent with the picture derived from molecular data [66,67].

### (e) Language study: Indo-European

The Indo-European languages—including English, Spanish, Hindi and Farsi—are spoken by around half of the world’s population as a first language [68]. The origins of this language family have been contested for over 200 years. The Steppe hypothesis proposes an expansion from the Pontic-Caspian Steppe (Eastern Europe and Central Asia) some time after 4500 BCE [69]. The farming hypothesis suggests that Indo-European dispersed from the Fertile Crescent (North Africa and Eurasia) around 6500–7500 BCE [70].

Heggarty *et al*. [71] compiled an extensive dataset containing 4990 cognates across 109 present-day and 52 ancient Indo-European languages [71]. The oldest languages (such as Hittite, Luvian and Mycenaean Greek) date to before 1000 BCE. Using a relaxed (gradual) clock model, they estimated that Indo-European dispersal started around 6150 BCE; an age inconsistent with both the Steppe and farming hypotheses. Rather, they propose a hybrid explanation. A recent study points out that this hypothesis is difficult to reconcile with genetic data [72].

We reinterpreted the Heggarty dataset by accounting for abrupt evolution. There was strong support for punctuated bursts of evolution, which can explain effects that gradualistic and covarion [73] processes were unable to. As shown in figure 5, the majority of recorded ancient languages are comparatively recent (e.g. younger than 1000 BCE). Consequently, the earlier lineages have more stubs (reflecting fewer samples), and therefore they conceal many hidden bursts of evolution. To reduce *a priori* analytical bias toward either the Steppe or farming hypotheses, we chose a tree prior distribution with an average height of 5100, and a 95% credible interval of 2760 -- 7700 BCE. An ‘epoch model’ [53,74] would overcome the limitations imposed by our assumption, not applied in the previous study, of homogeneous branching through time. Taken together, our results further corroborate the hybrid hypothesis. No doubt their model will remain a subject of debate by linguists in the years to come.

### (f) The parameter space is large but still tractable to explore

The gamma spike model clearly has a large parameter space. Each branch i is described by a time duration $\tau_{i}$, a relative clock rate $r_{i}$ and a spike sum $s_{i}$. For example, the aaRS tree contains 142 taxa and 282 branches; and therefore 282×3=846 tree parameters, and the topology itself. There are many ‘degrees of freedom’, but as Bayesian inference recognises, not all degrees are equally free.

While the tree’s parameter space is large, its terms are quite constrained; which keeps co-estimation tractable, and the *effective* degrees of freedom in check. The branch lengths τ are constrained by: (i) fixing leaf heights to the time they were sampled; and (ii) through the tree prior distribution, which further limits the range of probable branch lengths equation (2.1). The branch rates r are constrained through the relaxed clock standard deviation $\sigma_{r}$ equation (2.6), which is *a priori* assumed to adopt a small or moderate value ≈0.5*.* A log-normal distribution with a standard deviation of $\sigma_{r}=0.5$, for example, means the 95% interquartile range of branch rates spans a factor of 7.1; and $\sigma_{r}=0.4$ gives a factor of 4.8. Thus, as $\sigma_{r}$ approaches 0, the gradual evolution model approaches a strict clock where all branch rates are equal. Likewise, branch spike sums s are constrained through a gamma distribution with a shape of $S_{\alpha }$ equation (2.7), which is assumed to take on a value ≈2. For instance, a gamma distribution with shape $S_{\alpha }=2$ means that the 95% interquartile range of branch-spike-sums spans a factor of 23, if the branch has no stubs. With one stub, this factor becomes 8; and with two stubs it becomes 5.3. As $S_{\alpha }$ approaches infinity, the abrupt evolution model approaches a strict clock, where all spikes are equal. Enforcing branch times, rates, and spikes to adopt a narrow range of values, centred on some estimated average, ensures that these three terms can describe the evolutionary processes attributed to their respective explanatory model.

Indeed, our experiments confirmed that the effects of overparameterization are minimal. First, we demonstrated that data simulated under a gradual model is seldom supported by the spike model (figure 3D; electronic supplementary material, S4). This confirms that these extra parameters (the spikes) do not ‘overfit’ to random noise, even when no prior information is available. Second, we showed there are minimal levels of covariation between branch rate and spike estimates. Intuitively, a large-spike and a low-rate can explain the same process as a low-spike and a large-rate, and therefore we would expect to see a negative correlation between these two estimates. However, as table 1 and the electronic supplementary material, table S1 both show, these negative correlations are quite modest; with the most extreme Pearson correlation being $\rho_{sr}=-0.17$ (Indo-European). This further corroborates the notion that rates and spikes are capturing distinguishable processes; justifying the additional parameters. Lastly, we built upon an advanced pool of proposal kernels [41,46,50–53] to ensure an efficient traversal of this complex parameter space during MCMC. Although the time to achieve full convergence during MCMC is highly stochastic, most of the 14 empirical datasets analysed here converged in less than 3 h of MCMC runtime, with the larger datasets requiring up to two weeks.

## 4. Discussion

Saltative branching plays an important role in shaping the course of evolution across varying granularities of life, from genes to species to human civilizations. To investigate this phenomenon, we formulated a probabilistic framework for building phylogenies and testing for saltative branching in a single joint analysis, without the need for marginal likelihood approximation [75]. We demonstrated that this is a statistically consistent and reliable means of hypothesis testing, even when no prior information is available. As shown in figure 2, the model has a large parameter space. To remedy this concern, we: (i) constrained branch rates and spikes to adopt a narrow range of values, centred on some estimated average; (ii) employed Bayesian model averaging to disqualify extra parameters when they are not needed; and (iii) used state-of-the-art proposal kernels that traverse this multidimensional space effectively [41,46,50–53]. Our experiments all indicate that these efforts were successful. Given its complex nature, the model behaves surprisingly well on both simulated and empirical datasets.

In this view of evolution, one must consider lineages that are extinct or unsampled. These unobserved bifurcation events, or stubs, left behind footprints on those that did survive [23,37,43]. Such hidden speciation events, and therefore hidden bursts of abrupt evolution, are most prevalent among older lineages (figures 3 and 5). The view of gradualism, by contrast, is generally agnostic concerning the interplay between speciation and evolutionary change [3–6].

This work stands apart from existing methods. Pagel *et al*. provided a post-processing regression tool for testing punctuated evolution on a predetermined tree, which might not be built with abrupt evolution in mind [21]. Manceau *et al*. produced a method that jointly infers spikes and other parameters, while testing abrupt evolution on a branch-by-branch basis, but also on a fixed tree [23]. Most recently, Kaiping & Neureiter implemented a strict-abrupt relaxed-gradual clock model that jointly infers the tree [37]. Our approach combines and expands upon the best from these previous efforts, as it (i) enables testing for abrupt evolution, (ii) jointly infers the tree with the spike model, and (iii) uses the most general of clock models (figure 2). When the saltative branching hypothesis is rejected, the algorithm falls back on the relaxed clock [3,41], but with the added advantage of estimating stubs. Moreover, the Gamma spike model can use the full advantages of BEAST 2 [76], including the use of prior information to constrain the tree (e.g. to a single topology), and the inclusion of ‘empty sequences’ that account for taxa that have been discovered—but are lacking any data for analysis—so they can still inform the spike model. Our approach also differs from a recent model by Dieselhorst & Berg [26], which assumes that evolutionary change occurs exclusively at the time of branching (i.e. during cell division).

Understanding saltative branching can unravel powerful insights, as we demonstrated for the primordial differentiation of aminoacyl-tRNA synthetase enzymes, the diversification of cephalopods, and the dispersal of Indo-European languages. In each case, there was strong support for saltative branching. However, the impact was quite different in each scenario. First, this approach overcame biases when estimating divergence times and tree topologies, most notably in the aminoacyl-tRNA synthetases; probably reflecting their extensive structural and functional diversification over the past approximately 4 Ga [14,15]. Second, the (gradual) clock rate is slower when abrupt evolution is accounted for (table 1), especially the cephalopod clock rate, estimated to be two orders of magnitude slower. Remarkably, 99% of their morphological changes were found to occur at the time of speciation, suggesting their morphological evolution was almost exclusively governed by sudden bursts of adaptive radiation as the animals populated new marine niches. This might also reflect dependencies between morphological traits (such as the shape of a shell, and the presence/absence of a shell), which change together in rapid sweeps rather than through independent diffusion. Third, spike magnitudes varied considerably; with the aaRS (and cephalopods) seeing 1 in 15 (22) amino acids (traits) change at each bifurcation event, but Indo-European languages just 1 per 700. The magnitudes of these estimates, as well as those in standard benchmark datasets (electronic supplementary material, table S1; DS1−S11 [56]), are in line with previous studies [21,23,37]. However, small spikes do not diminish the impact of saltative branching—for example 24% of all changes in Indo-European languages were estimated to occur at the time of branching, despite undergoing an average of just 0.001 changes per cognate per bifurcation. Accounting for abrupt evolution does not always alter the overall conclusion to be drawn, in which case it establishes robustness; as it did for estimating the origin of Indo-European languages.

There exist numerous avenues for future investigation. This approach offered idiosyncratic outcomes in each case study, and may behave differently yet again in other settings; potentially illuminating new modes of saltation. This study assumed that speciation and extinction occur homogeneously through time, however an enhancement would involve varying these processes across epochs [74] or among lineages [77]. Future investigations might also explore the possibility of further integrating branching and evolution, for instance, by incorporating stub-awareness into relaxed or local models of gradual evolution [3–6], coalescent models including the multispecies coalescent [8,78], or at the task of species delimitation [79] or ancestral reconstruction [13]. Taken together, this work places us one step further from treating evolutionary change as a paint bucket, and one step towards a sculpting chisel.

## Data Availability

Our General Public Licensed source code is available as the GammaSpikeModel package for BEAST 2, where it is readily configured through a user-friendly interface. XML files, source code, and documentation is available on GitHub at [80], with version 1.0.0 archived on Zenodo at [81]. Posterior distribution log files for our three case studies are available on Dryad at [82]. Supplementary material is available online [83].
